# Evolutionary bi-level neural architecture search with training: A framework for color classification

**DOI:** 10.1038/s41598-025-22538-6

**Published:** 2025-11-04

**Authors:** Mitchell Ángel Gómez-Ortega, Miguel Gabriel Villarreal-Cervantes, Mario Aldape-Pérez, Alam Gabriel Rojas-López, Daniel Molina-Pérez, Ramón Silva-Ortigoza

**Affiliations:** 1 Instituto Politécnico Nacional, RERYM, CIDETEC, Ciudad de México, 07700 México; 2 Instituto Politécnico Nacional, ESCOM, Ciudad de México, 07700 México

**Keywords:** Neural network, Bi-level optimization, Evolutionary optimization, Neural architecture search, Information technology, Electrical and electronic engineering

## Abstract

The design of Artificial Neural Networks (ANNs) for classification tasks has been a topic of interest. However, defining an optimal ANN architecture remains challenging, especially when considering resource constraints and the large number of design parameters. This paper proposes an Evolutionary Bi-Level Neural Architecture Search with Training (EB-LNAST) approach that simultaneously optimizes the architecture, weights, and biases of a neural network using a bi-level optimization strategy. The upper level focuses on minimizing the network complexity penalized by the lower level performance function, while the lower level optimizes training parameters to minimize the loss function and maximize the predictive performance. The proposal is evaluated on a real-world color classification task and the WDBC dataset, demonstrating statistically significant improvements over traditional machine learning algorithms, as well as advanced models. Compared to Multilayer Perceptron (MLP) based algorithms, EB-LNAST achieves superior predictive performance when the architecture is fixed, and remains competitive, with a marginal reduction in performance of no more than $0.99\%$, even when compared against MLPs optimized with extensive hyperparameter tuning, including architecture, activation functions, regularization, and optimizers. Remarkably, EB-LNAST achieves up to a $99.66\%$ reduction in model size, highlighting its ability to discover compact and efficient architectures. EB-LNAST is a reliable alternative for generating compact and effective neural network architectures in accordance with the problem’s requirements, enabling efficient exploration of the search space while maintaining or exceeding the predictive performance of state-of-the-art classification algorithms.

## Introduction

### Background and motivation

Artificial Neural Networks (ANNs) are one of the most well-known techniques in artificial intelligence, with the primary goal of enabling machines to make decisions, perform analytical evaluations, and make comparative judgments, thereby simulating human behavior. ANNs are methods inspired by the biological functioning of the human brain^1^. During the past decades, ANNs have become popular for solving problems in various fields of knowledge, for instance, in color classification^2^, textile industry monitoring^3^, color classification in tempering processes^4^, air overpressure prediction^5^, and vibration analysis caused by mining explosions^6^. In the medical field, applications range from X-ray image analysis^7^ to energy-related applications^8^, as well as studies on Convolutional Neural Networks (CNNs).

In most cases, ANNs are trained using the backpropagation method, which adjusts weights and biases to minimize prediction error through gradient descent^9^. In spite of backpropagation being widely used for weight optimization in feedforward neural networks (FNNs), it may present limitations in specific training scenarios, such as a tendency to become trapped in local minima. Various factors can hinder convergence and may lead to overfitting, including the initial network conditions, learning rate, and the inherent complexity of the problem, among others. To mitigate these issues, regularization techniques such as L2 (Ridge Regularization), L1 (Lasso Regularization), Dropout, and batch normalization are frequently employed to improve the generalization of the ANN performance by reducing error and increasing the likelihood of reaching a global minimum^10^.

Another issue that plays a vital role in network performance lies in the architecture design. The ANN architecture design is another critical and complex aspect that involves determining the number of layers, the number of neurons per layer, layer types, connection schemes, and other factors. As the ANN training requires a fixed and predefined architecture, the combined selection of the training and architecture parameters is neither intuitive nor systematic^11^, such that designers require extensive experience because improper selection of design parameters can lead to loss of ANN performance^12–16^.

On the other hand, metaheuristic algorithms have emerged as robust alternatives for training artificial neural networks (ANNs) and for finding optimal network architectures, addressing this challenge^17^. These algorithms are based on heuristic principles inspired by natural phenomena, physical processes, or collective intelligence strategies to strike a balance between the exploration of new solution space areas and the exploitation of known solutions to refine them^18^. Various metaheuristic algorithms inspired by biological evolution enhance the search for solutions, guiding convergence to the closest region of the global minimum and overcoming the previously mentioned limitations. The most commonly used methods include evolutionary algorithms (EAs), which optimize network structure and weights, as well as other parameters, by employing mutation and crossover operators to explore the solution space efficiently. In^19^, the Particle Swarm Optimization (PSO) algorithm is employed to optimize the training parameters, such as weights and biases of the ANN, thereby balancing the exploration and exploitation of the search space. Evolutionary programming is used to simultaneously combine the network architecture and connection weights to find optimal neural network configurations^20^.

The use of deep neural network architecture search techniques has also been explored to improve the output of the neural network. For instance, the medical image segmentation is improved by optimizing convolutional neural network architectures using the genetic algorithm^21^. On the other hand, an auto-adaptive approach is used for architecture search employing the metaheuristic Teaching-Learning-Based Optimization (TLBO)^22^. Architecture search is also addressed through an evolutionary multiobjective approach based on supernets^23^. Evolutionary search for monocular depth estimation and image classification is also addressed in^24^, and the Warm-Start Multiobjective Evolutionary Algorithm is employed for graph neural networks in^25^. Alternatively, other techniques for Neural Architecture Search (NAS) include Random Search, Q-Learning, and Bayesian Optimization, which are used to explore and optimize the architecture of Deep Neural Networks in drug response prediction^26^. The use of regularization techniques aids in preventing overfitting and improving model generalization. Reinforcement learning in graph assembly^27^ is another strategy, as well as the use of Differentiable Architecture Search (DARTS) to reduce the spatial redundancy of features in computer vision techniques that facilitate feature learning^28^. These examples illustrate active research in the field of searching for efficient architectures for deep learning, where optimization is not limited to training parameters but extends to the very design of the network. The diversity of the search approaches underscores the importance of finding optimal architectures as a fundamental step towards improving the performance in specific applications, which in turn influences the need for appropriate parameter optimization for each architecture found.

Every parameter that affects the performance of an ANN is considered a HyperParameter (HP). These hyperparameters can be categorized into five main categories depending on the aspect of the network they impact. The first group is related to the architecture parameters, which define the network structure relating to the layer numbers, activation functions, network type, etc. The second group includes training parameters that adjust the learning process, such as the learning rate, batch size, epoch number, and optimizers. The regulation parameters involve the third group, which aims to prevent overfitting and includes L1/L2 weight penalties, dropout, and other methods. The fourth group is related to the preprocessing parameters that influence the input data quality, such as normalization and augmentation strategies. The fifth group is directly related to the particular characteristics of each type of neural network. For instance, in convolutional neural networks, the hyperparameters could be related to the filter size, stride, and padding; in both recurrent neural networks like Long Short-Term Memory (LSTM) and Bidirectional Long Short-Term Memory (Bi-LSTM) those parameters are related to the number of memory units, the recurrent dropout rate; in Transformer networks the HPs include the number of attention heads, the number of encoder and decoder layers, among others. Defining the appropriate values is a challenge, which is why the process of determining suitable parameters is referred to as Hyperparameter Optimization (HO)^29^.

Regarding optimization techniques^30^, presents an analysis of HO techniques in deep learning applications, addressing challenges in applying these techniques and comparing different algorithms. This analysis covers key hyperparameters related to model training and structure, such as learning rate, batch size, optimizer, number of hidden layers, and layer width, discussing their importance and methods for defining their value ranges. The study evaluates the efficiency and accuracy of gradient-based optimization algorithms, including Stochastic Gradient Descent (SGD) and its variants, such as AdaGrad, RMSProp, and Adam, particularly in the context of deep learning networks. While the review provides a comprehensive overview of HO algorithms, tools, and applications, it acknowledges limitations, such as the performance of classical search algorithms for large hyperparameter spaces and the computational expense of evaluating complex deep learning models. Similar studies on hyperparameter optimization^31–34^ focus on tuning parameters, such as learning rate, number of hidden layer nodes, number of filters, LSTM network dimensions, number of training epochs, L2 regularization technique, dropout rate, and entropy coefficient, among others, with the use of gradient-based algorithms.

According to the aforementioned works, hyperparameter optimization is a critical factor. In^35^, the Robust and Efficient Hyperparameter Optimization at Scale (BOHB) approach is utilized. This method combines Bayesian Optimization (BO) with bandit-based evaluation strategies, such as Hyperband (HB), to achieve a balance between performance and rapid convergence toward optimal configurations for various types of Artificial Neural Networks (ANNs). The considered hyperparameters (HPs) are learning rate, momentum, batch size, and regularization techniques such as Weight Decay and Dropout rates.

The application of metaheuristic algorithms in the Deep Learning framework includes implementations of PSO, Ant Colony Optimization (ACO), Firefly Algorithm (FFA)^36^, the Dragonfly Algorithm^37^, Sparrow Search Algorithm (SSA)^38^, and multiobjective Swarm Algorithm (MSA)^39^ to address kernel size, layers, convolutional layers, pooling layers, among others.

Beyond the optimization of hyperparameters, the architecture of a neural network itself plays a fundamental role in its performance. A case study addressing ANN architecture optimization is presented in^40^, where a Convolutional Neural Network (CNN) is optimized regarding convolutional layers, the number of nodes per layer, and filter size using Differential Evolution (DE) to identify hyperparameters that compete with state-of-the-art CNNs. Other studies, such as those presented in^41^ and^42^, also address the optimization of ANN architectures, considering aspects such as the number of layers, the number of hidden nodes, and the number of hidden layers. To simultaneously optimize multiple hyperparameters, bi-level optimization^43^ can be considered a solution. This hierarchical problem-solving technique operates with two independent optimization levels. The upper level controls decisions that condition those of the lower level, which resolves a subordinate problem based on upper level decisions. By addressing the optimization problem in a bi-level manner, the possibility arises to define and optimize a much broader set of hyperparameters jointly. In^44^, the authors explored neural architecture search (NAS) for graph neural networks (GNNs) using a framework called Adaptive Bayesian Genetic Neural Architecture Search (ABG-NAS). This approach extends evolutionary methods by incorporating a comprehensive search space of propagation and transformation operations, specifically designed for learning graph representations. Another relevant method is DDS-NAS, proposed in^45^, which integrates dynamic data selection (DDS) into the NAS process. By combining hard example mining with curriculum learning, DDS-NAS constructs balanced training subsets that are dynamically updated as the model masters simpler examples, replacing them with increasingly challenging and diverse samples from the same class. A further contribution is DEEP Q-NAS, presented in^46^, which introduces a reinforcement learning–based NAS scheme employing Deep Q-Networks (DQNs). Those works focus on NAS as a means to overcome the labor-intensive nature of manual architecture design and the dependence on specialized expertise. Nevertheless, in the context of automatic architecture search, those recent approaches share a scheme to direct attention to the search of architecture parameters (dimension size, topology, and so on) that maximizes of the model predictive performance on a validation set, either through metrics such as F1-scores, accuracy, or precision, while adjusting the training parameters (weights, biases, optimizer setting, and so on) by minimizing the loss function as the objective function, either through metrics such as cross-entropy, Mean Square Error (MSE). So, those approaches focus exclusively on improving predictive performance, which may lead the search process to unnecessarily complex architectures because they can not penalize overparameterized models, resulting in computationally expensive architectures and impractical for execution in systems with limited computing resources.

### Contributions

In contrast to previous approaches, this work introduces a synergetic neural architecture search framework based on evolutionary bi-level optimization where in the upper level, the method implements an optimization strategy that explicitly balances predictive accuracy with architectural simplicity by minimizing structural complexity (i.e., number of layers and neurons) while penalizing inadequate validation performance, as quantified by the $F_{\beta }$ metric, and jointly integrating the training MSE given in the lower lever. In this way, the upper-level objective is designed not only to achieve high predictive performance but also to enforce compactness and stability in the resulting model. At the lower level, the optimization process simultaneously considers validation $F_{\beta }$ and training MSE for updating weights and biases, thereby ensuring that the evaluation metrics are consistently aligned with the optimization objectives. Unlike most studies reported, which often emphasize only predictive performance or rely on a single optimization criterion, the proposed framework explicitly integrates both predictive and architectural efficiency in a unified bi-level formulation. This dual focus constitutes a key novelty of the present work and differentiates it from existing approaches in the field.

To the best of the author’s knowledge, this is the first work that simultaneously optimizes the architecture, weights, and biases of ANNs in a general context using a bi-level optimization strategy with the aim of reducing the complexity of the network while achieving predictive performance comparable to, or better than, more complex models. As the case study, the proposed approach is applied to a color classification problem and to a complex dataset.

Then, the main contributions of this work are threefold. First, it introduces a novel approach called Evolutionary Bi-Level Neural Architecture Search with Training (EB-LNAST), which simultaneously searches for both the optimal model architecture and its parameters. This bi-level optimization framework leads to a more efficient search process, mitigates overfitting to specific configurations, and results in models that are not only more compact but also demonstrate improved generalization performance compared to traditional methods. Second, the effectiveness and robustness of the proposed method are demonstrated through its application to a real-world problem and a widely used complex benchmark dataset, providing empirical evidence of its practical utility. Third, a comparative performance analysis is conducted against several state-of-the-art machine learning algorithms, with statistical validation confirming that EB-LNAST consistently outperforms or remains competitive with existing methods and offers measurable advantages in terms of both accuracy and model efficiency.

**Fig. 1:**
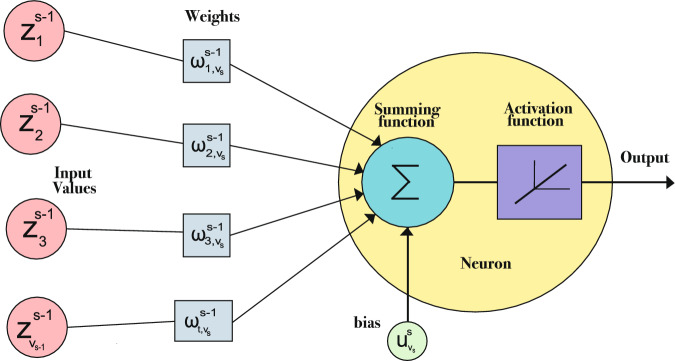
Detail of the node in the MLP from Figure 2.

The remaining structure of this work is organized as follows: The first Section details the proposed bi-level Optimization approach applied to the design and optimization of neural network architectures. The second Section presents the specific case study of color classification, detailing the problem addressed and the system used for experimentation. In the third Section, the results obtained by applying the proposed approach are presented and analyzed, including the initial conditions of the optimizers, a performance analysis of the bi-level method, and its comparison with other machine learning approaches and another well-known dataset. The final Section presents the main conclusions derived from the research, highlighting the key findings and contributions of the work. This final section also outlines possible future research directions and limitations identified in the present work. (Fig. 1)

## Bi-level optimization approach in neural network architecture

**Fig. 2:**
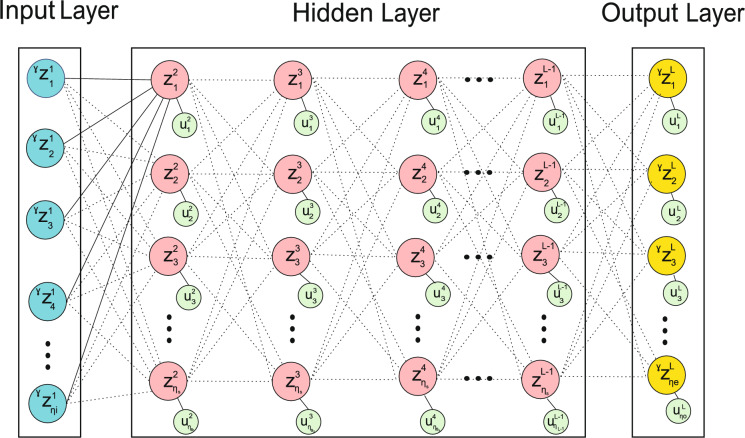
Multi-layer neural network.

The Evolutionary Bi-Level Neural Architecture Search with Training (EB-LNAST) approach addresses two common problems in ANNs: architecture search, training, and validation, with a focus on different architecture sizes. In the EB-LNAST approach, it is assumed that the ANN architecture takes the structure of a multi-layer perceptron (MLP), as shown in Figure 2. The MLP is a type of artificial neural network composed of multiple layers of neurons, with additional hidden layers between the input and output, each containing nodes^47^. The characteristic equation representing a node is given in (1) and shown in Figure 1. The overall MLP architecture is displayed in Figure 2.

1 $$\begin{aligned} z^s_{v_s} = f \left( \sum _{t=1}^{|v_{s-1}|} \omega ^{s-1}_{t,v_s} \cdot z^{s-1}_{v_{s-1}} + u^s_{v_s}\right) \end{aligned}$$

where

- $s = \{1, 2, \dots , L\} \in \mathbb {Z}^+$: Represents the index of the $s$-th layer of the neural network, where $L$ is the total number of layers.
- $v_s \in \mathbb {Z}^+$: Represents the index of a neuron (node) within the $s$-th layer. The general relation is described in (2).
  2 $$\begin{aligned} v_s = {\left\{ \begin{array}{ll} v_r = \{1, 2, \dots , \eta _i\}, & \text {if } s = 1, \\ v_m = \{1, 2, \dots , \eta _{h_s}\}, & \text {if } s \in \{2, \dots , L-1\}, \\ v_q = \{1, 2, \dots , \eta _o\}, & \text {if } s = L. \end{array}\right. } \end{aligned}$$
  The value of $s$ depends on the layer:
  - For the input layer ($s = 1$): $v_s = v_r = \{1, 2, \dots , \eta _i\}$, where $\eta _i$ is the maximum number of neurons in the input layer.
  - For the hidden layers ($s = \{2, \dots , L-1\}$): $v_s = v_m = \{1, 2, \dots , \eta _{h_s}\}$, where $\eta _{h_s}$ is the maximum number of neurons in the $s$-th hidden layer.
  - For the output layer ($s = L$): $v_s = v_q = \{1, 2, \dots , \eta _o\}$, where $\eta _o$ is the maximum number of neurons in the output layer.
- $z^s_{v_s} \in \mathbb {R}:$ Represents the output of the neuron $v_s$ in the $s$-th layer, including the activation function:
  - For the input layer ($s = 1$), ${}^{\gamma } z^1_{v_r}$ corresponds to the input data of the $v_r$-th neuron. The term $\gamma$ represents the $\gamma$-th input of the neuron $v_r$.
  - For the hidden layers ($s \in \{2, \dots , L-1\}$), $z^s_{v_m}$ is the output of the $v_m$-th neuron in the $s$-th hidden layer.
  - For the output layer ($s = L$), ${}^{\gamma } z^L_{v_q}$ is the output of the $v_q$-th neuron. The term $\gamma$ represents the $\gamma$-th output of the neuron $v_q$ in the output layer $L$ for the $\gamma$-th input vector ${}^{\gamma } z^{1}_{v_r}$ $\forall$ $v_r$.
- $u^s_{v_s} \in \mathbb {R}$: Represents the bias of the neuron $v_s$ in the $s$-th layer. It is worth mentioning that for the input layer, no bias is included ($u^1_{v_s} = 0$).
- $\omega ^{s-1}_{t, v_s} \in \mathbb {R}$: Expresses the synaptic weight that connects the $t$-th neuron in the $s-1$-th layer with the $v_s$-th neuron in the $s$-th layer (for $s> 1$).
- $f(z^{s-1}_{v_{s-1}}, \omega ^{s-1}_{t, v_s}, u^s_{v_s})$: Represents the activation function, which defines the transformation applied to the output of the neuron $z^{s}_{v_s}$.

**Fig. 3:**
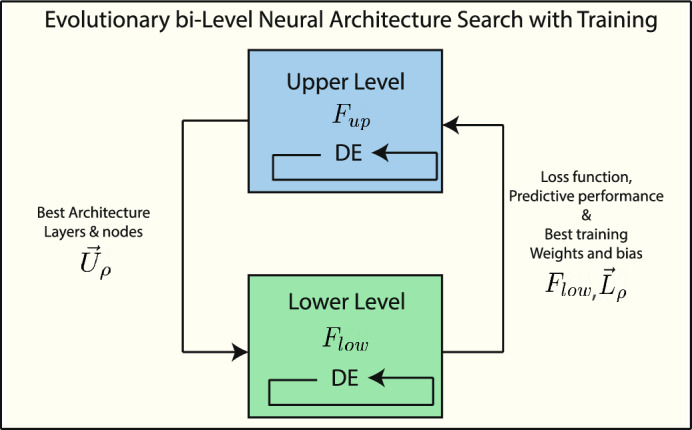
Graphical representation of the upper and lower levels in the proposed EB-LNAST approach.

For the EB-LNAST approach, two interdependent decision-making levels are considered, i.e., with two levels of upper and lower optimization. Fig. 3 displays a graphical representation of the proposed approach with the corresponding interactions between layers. In the upper level, the goal is to find the minimal MLP architecture. In the lower level, the goal is to optimize the weights and biases of the architecture from the upper level using a simultaneous training and validation strategy. If the architecture size is modified, the number of weights and biases could increase or decrease.

### Upper level: architecture design

In terms of architecture, it is known that the input $^{\gamma }z_{v_r}^{1}$ is defined by the data provided by the user to the MLP. Similarly, the output data $^{\gamma }z_{v_q}^{L}$ is also set from the beginning. Therefore, the variables to be determined in the upper level are the total number of hidden layers, defined as $h_L$, and the number of neurons (nodes) corresponding to each hidden layer. Thus, the total number of layers *L* is given by $L=h_L + 2 \in \mathbb {Z^+}$.

The upper optimization problem consists of determining the minimal architecture, which includes the number of hidden layers $h_L$ and the number of nodes in each hidden layer, described as $\eta _{h_s}$ $\forall$ $s=\{2,...,L-1\}$, that generate the maximum validation of the input data of the MLP performance. Therefore, the variables mentioned above are considered as the upper level design variables $\vec {U}_\rho =[\vec {U}_{\rho _{1}},\vec {U}_{\rho _{2}}]^T \in \mathbb {N}^{1 + \eta _{{h_2}}+ \ldots +\eta _{h_{L-1}}}$, which are grouped in (3), where $\vec {U}_{\rho _{1}}$ relates the hidden layer numbers and $\vec {U}_{\rho _{2}}$ includes the node number of each hidden layer.

3 $$\begin{aligned} \vec {U}_\rho = [h_L, \eta _{h_2}, \ldots ,\eta _{h_{L-1}}]^T \end{aligned}$$

Once the upper-level design variables are defined, the first term $J_{{up}_{1}}( \vec {U}_{\rho })$ of the objective function for the upper level $F_{up}$, represented in (4), is formulated. This level examines the MLP architecture in terms of its hidden layers and the nodes within each hidden layer.

4 $$\begin{aligned} J_{{up}_{1}}( \vec {U}_{\rho }, \vec {L}_\rho )= h_{L} + \sum _{s=2}^{L-1} \eta _{h_s} \end{aligned}$$

At this upper level, a constraint is established such that the design variables from the lower level, $\vec {L}_\rho$, solve an optimization problem related to the training and validation of the MLP, resulting in the MLP’s weights and biases. Therefore, at this upper level, the lower-level design vector $\vec {L}_\rho$ is considered constant.

The second term of the upper-level objective function is presented in (5). This second term includes the training and validation of the ANN through the Mean Squared Error (MSE) and $F_\beta$^48^ metrics, which are provided by the objective function of the lower level $F_{low}(\vec {U}_{\rho }, \vec {L}_\rho )$. Therefore, these metrics incorporate a constant penalty for the upper-level objective function, which is proportional to the training and validation error obtained at the lower level. This second term is explained in detail in Section 2.2.

5 $$\begin{aligned} J_{{up}_{2}}(\vec {U}_{\rho },\vec {L}_\rho ) = F_{low}(\vec {U}_{\rho },\vec {L}_\rho ) \end{aligned}$$

Therefore, to define the upper-level objective function (6), it is necessary to combine (4) and (5) using the weighting coefficients $a_{s_1}$ and $a_{s_2}$, which determine the importance of each element within the objective function during the optimization process.

6 $$\begin{aligned} F_{up}(\vec {U}_{\rho },\vec {L}_{\rho })=a_{s_1} J_{{up}_{1}}( \vec {U}_{\rho },\vec {L}_{\rho }) + a_{s_2}J_{{up}_{2}}(\vec {U}_{\rho },\vec {L}_{\rho }) \end{aligned}$$

### Lower level

The design variables associated with the lower level modify the training and validation of the MLP. Therefore, the weights $\omega ^{s-1}_{t,v_{s}}$ and biases $u^{s}_{v_s}$ are considered variables to be determined at the lower level. These variables are grouped in $\vec {L}_\rho =[\vec {L}_{\rho _1},\vec {L}_{\rho _2}]^T \in \mathbb {R}^{(\eta _i \times \eta _{h_i}) + \sum ^{L-2}_{s=1} (\eta _{h_s} \times \eta _{h_{s-1}}) + (\eta _{h_{L-1}} \times \eta _o) + \sum ^{L-1}_{s=2} (\eta _{h_s}) + \eta _o}$, as shown in (7). $\vec {L}_{\rho _1}$ includes the weight values and $\vec {L}_{\rho _2}$ denotates the bias values.

7 $$\begin{aligned} \vec {L}_\rho =[\omega ^{s-1}_{t, v_s},u^{s}_{v_s}]^T \forall s=\{2,...,L\} \end{aligned}$$

The first term of the lower-level objective function is associated with the MSE of the MLP training, as shown in (8). The MSE minimizes the error between the target output $^{\gamma }\hat{z}^{o}_{v_q}$ and the output obtained $^{\gamma }z^{L}_{v_q}$ by the MLP during the optimization process, considering the $N_t$ input vectors ${}^{\gamma } z^{1}_{v_r}$ $\forall$ $\{v_r,\gamma \}$.

8 $$\begin{aligned} J_{{low}_{1}}(\vec {U}_{\rho },\vec {L_\rho })= \frac{1}{|v_q|} \sum _{\gamma =1}^{N_t} \sum _{v_q} ( ^{\gamma }z^{L}_{v_q}-^{\gamma }\hat{z}^{o}_{v_q} )^2 \end{aligned}$$

The second term of the lower-level objective function considers the MLP validation. For this, a set of inputs provided to the MLP must be regarded. This set can be divided into $N_t$ training data and $N_v$ validation data, as reducing the MSE during MLP training does not guarantee suitable generalization. In fact, the MLP may fail to perform appropriately on unknown data during the test phase. For this reason, the second objective function is established as the $F_{\beta }$ metric, described in (9). This metric enables the evaluation of MLP performance, particularly in binary classification problems, as it combines Precision and Recall through a factor $\beta$ that adjusts the balance between the two measures when assessing the network’s performance. Precision provides information about the model’s ability to identify positive cases, while Recall quantifies the proportion of true positive cases correctly identified. Combining them reduces false positives and, simultaneously, decreases false negatives^49^. Furthermore, the values of $F_\beta$ lie within the range [0, 1], where a value of 1 indicates $100\%$ prediction accuracy, while a value of 0 corresponds to $0\%$ prediction accuracy.

9 $$\begin{aligned} \begin{aligned} J_{{low}_{2}}(\vec {U}_{\rho },\vec {L\rho })&=F_{\beta } \\&= (1+ \beta ) \frac{(Precision)(Recall)}{(\beta ^2 (Precision)+Recall)} \end{aligned} \end{aligned}$$

In expression (9), Precision and Recall are defined in equations (10) and (11), respectively. True Positives (TP) refer to cases where the model correctly predicts a positive instance when it is indeed positive in the data. False Positives (FP) are cases where the model incorrectly predicts a positive instance when it is negative. True Negatives (TN) refer to instances that are correctly predicted as negative, while False Negatives (FN) occur when the model incorrectly predicts a negative outcome for a truly positive instance. To better visualize the distribution of correct and incorrect predictions across multiple classes, a multiclass confusion matrix is employed in the proposed approach^50,51^.

10 $$\begin{aligned} & Precision = \frac{TP}{TP + FP} \end{aligned}$$

11 $$\begin{aligned} & Recall = \frac{TP}{TP + FN} \end{aligned}$$

To formulate the lower-level objective function (12), it is necessary to unify the terms associated with training (8) and validation (9) using a weighted sum approach. The weighted sum approach assigns the weighting coefficients $a_{1_{low}}$ and $a_{2_{low}}$ to each term. At this lower level, the goal is to minimize the MSE ($J_{{low}_{1}}(\vec {L}_\rho )$) and maximize the $F_\beta$ metric ($J_{{low}_{2}}(\vec {L}_\rho )$), so the negative sign is assigned to $J_{{low}_{2}}(\vec {L}_\rho )$ to formulate the optimization problem as a minimization one.

12 $$\begin{aligned} F_{low}(\vec {U}_\rho ,\vec {L_{\rho }})= a_{1_{low}} J_{{low}_1}(\vec {L_{\rho }}) - a_{2_{low}} J_{{low}_2}(\vec {L_{\rho }}) \end{aligned}$$

### General formulation of the EB-LNAST optimization problem

Once the design variables, objective functions, and constraints of the EB-LNAST approach are defined, the optimization problem is formulated to find the optimal trained architecture that considers the input and output data, thereby achieving a suitable trade-off between training and validation of the MLP. Therefore, the general formulation of the proposed optimization problem is shown in (13).

13 $$\begin{aligned} \begin{aligned}&\min _{\vec {U}_\rho , \vec {L}_{\rho }} F_{up}(\vec {U}_{p},\vec {L}_{\rho }) \\&\text {s.t.} \\&\quad \vec {U}_\rho \in \underset{\vec {L}_\rho }{\operatorname {argmin}} \left\{ \begin{array}{ll} F_{low}(\vec {U}_{\rho },\vec {L_{\rho }}) \\ s.t. \\ \vec {L}_{\rho _{min}} \le \vec {L}_{\rho } \le \vec {L}_{\rho _{{max}}} \end{array} \right. \\&\quad \vec {U}_{\rho _{min}} \le \vec {U}_{\rho } \le \vec {U}_{\rho _{max}} \end{aligned} \end{aligned}$$

### Optimization technique

Traditionally, neural network training is performed using the backpropagation algorithm^9^, a widely used method due to its simplicity and efficiency. However, its main drawback is the possibility of getting trapped in local optima without guaranteeing convergence to the global optimal solution, which can negatively impact the model’s performance.

To address the optimization problem using the EB-LNAST approach, a bi-level model is proposed to overcome the limitations of the backpropagation algorithm. Since the upper-level objective functions involve discrete variables, multiple solutions must be generated instead of relying on gradient-based methods. In this work, DE, a global optimization algorithm inspired by biological evolution, is employed. DE enables a broader exploration of the solution space, facilitating the search for optimal architectures and ensuring the reproducibility of experiments. Additionally, it reduces the risk of getting trapped in local optima, offering a more versatile alternative for training artificial neural networks^52^.

To implement the EB-LNAST approach with DE, the variable ${\textbf {X}}_{sup}=[h_L, \eta _{h_2}, \ldots ,\eta _{h_{L-1}}]^T$ is assigned for the upper level, noting that at this level the variables are handled as positive integers. Consequently, for the lower level, the variable ${\textbf {X}}_{low}=[\omega ^{s-1}_{t, v_s}, u^{s}_{v_s}]^T$ is assigned with real values. The structure of the DE algorithm for the EB-LNAST approach is presented in Algorithm 1, specifically using the best/1/bin variant^53^.

**Algorithm 1:**
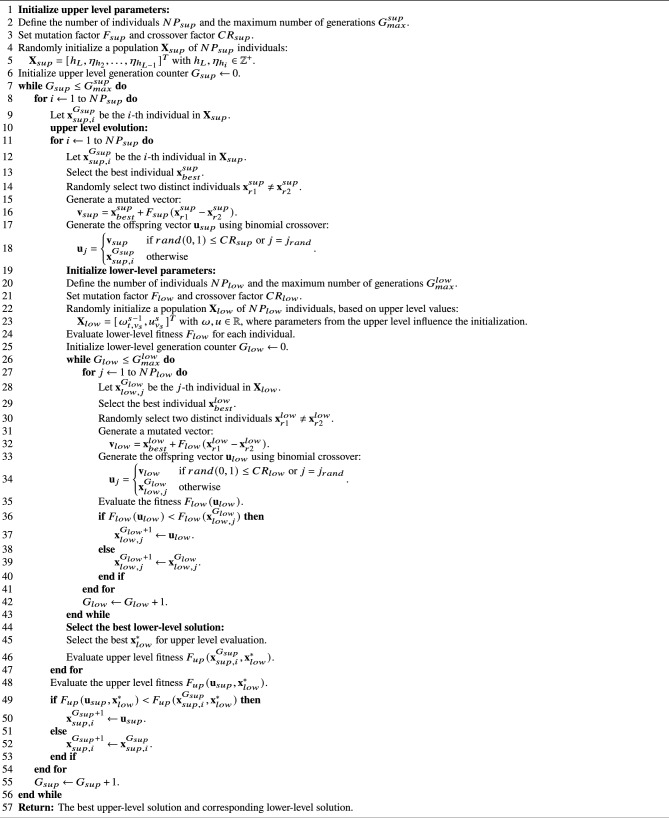
Differential evolution algorithm for bi-level optimization

In the related literature, many approaches to optimizing hyperparameters and neural network architectures rely on deterministic methods for training. However, when simultaneously searching for both network architectures and optimizing weights and biases, DE provides a robust framework by exploring multiple architectural solutions. This approach mitigates the issue of repetitive training, allowing for a more comprehensive exploration of potential solutions.

## Case study: color classification

As a case study for the EB-LNAST approach, the color classification through the ColorPal device from Parallax is included. The device features a compact *TSL13T* sensor that detects the intensity of light reflected by the object in millivolts. This voltage provides information about the light spectrum in which the object’s color is located. However, ambient light can introduce values that do not correspond to the actual light spectrum, making color identification a complex task.

Using the ColorPal device, a system is integrated with five 3D-printed tablets of different colors. The device is placed in a semi-enclosed base at a distance of 1 cm from the position where the tablets are located, aiming to measure light reflection in millivolts. The light reflections of the onboard RGB Light Emitting Diode (LED) are used as a distinctive feature (attribute) for color classification. Then, the device sequentially illuminates the tablet with red, green, blue, and white lights to form the input instance of the MLP network. The ambient light is also included in the input instance when the lights of the RGB LED are turned off. The sensitivity of ambient light is reduced by taking samples in different locations and times of the day.

**Fig. 4:**
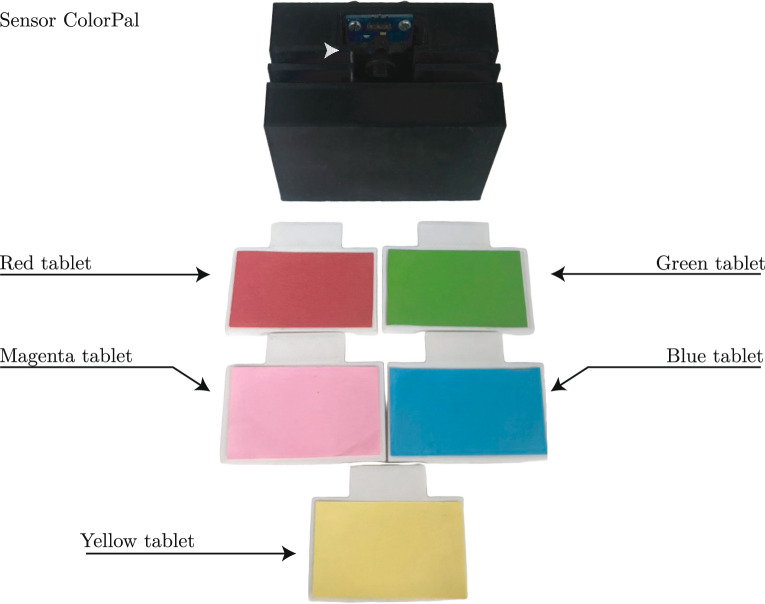
ColorPal system for measuring light reflection.

The ColorPal system, as shown in Figure 4, is designed to provide information about the input features related to the light reflections using five different light intensities. The EB-LNAST approach determines the optimal ANN architecture for identifying the color class to which an input instance belongs. The following section presents the particularities of the EB-LNAST approach in this case study.

### Optimization problem formulation for color classification

#### Input data

A total of 570 measurements (reflected light intensity-related voltages) are collected from 3D-printed tablets with colors cyan, red, yellow, green, and magenta under varying ambient light conditions. In this work, a sample consists of five different voltages related to the reflected light intensities on a specific color tablet. Fourteen samples using a color tablet with different ambient light are averaged to serve as an input instance (input data in the ANN). In total, seventy measurements are used to provide five instances or samples (five input data related to each color tablet) in the training of the EB-LNAST approach delivering a balanced data set. The mean reflected light intensities, in millivolts, corresponding to the five input instances are presented in Table 1.

**Table 1:** Input instances (input data) from the average of 14 measurements in *mV* obtain by ColorPal system.

			Color tablets				
			Cyan ($\gamma =1$)	Red ($\gamma =2$)	Yellow ($\gamma =3$)	Green ($\gamma =4$)	Magenta ($\gamma =5$)
Light intensities	Red	${}^{\gamma } z^{1}_{1}$	1358.07	1956.00	2716.50	785.36	2649.29
	Green	${}^{\gamma } z^{1}_{2}$	1643.64	493.93	2052.71	1152.36	1304.79
	Blue	${}^{\gamma } z^{1}_{3}$	3131.14	653.57	2219.36	897.21	2548.50
	White	${}^{\gamma } z^{1}_{4}$	1893.21	461.71	1325.93	753.00	1201.14
	Ambient	${}^{\gamma } z^{1}_{5}$	644.00	272.71	405.86	323.21	384.64

The input vector for the $\gamma$-th color tablet is presented as a vector in (14).

14 $$\begin{aligned} {}^{\gamma } z^{1}_{v_r} = [{}^{\gamma } z^{1}_{1}, {}^{\gamma } z^{1}_{2}, {}^{\gamma } z^{1}_{3}, {}^{\gamma } z^{1}_{4}, {}^{\gamma } z^{1}_{5}]^T \end{aligned}$$

For the validation process in the proposed approach, the previously mentioned 70 measurements (i.e., 14 samples) are used. The remaining 500 measurements (100 samples) are then employed to test the optimal architecture obtained.

#### Output data

The output is encoded using One-Hot Encoding, representing the target color in the tablet to be identified. Therefore, the MLP output for this case study is represented as a vector as in (15), where the corresponding MLP output vectors to each color tablet are presented in Table 2.

15 $$\begin{aligned} {}^{\gamma } \hat{z}^{o}_{v_q} = [{}^{\gamma } \hat{z}^{o}_{1}, {}^{\gamma } \hat{z}^{o}_{2}, {}^{\gamma } \hat{z}^{o}_{3}, {}^{\gamma } \hat{z}^{o}_{4}, {}^{\gamma } \hat{z}^{o}_{5}]^T \end{aligned}$$

**Table 2:** One-Hot Encoding for MLP Outputs.

Output	Color tablets				
	Cyan ($\gamma =1$)	Red ($\gamma =2$)	Yellow ($\gamma =3$)	Green ($\gamma =4$)	Magenta ($\gamma =5$)
${}^{\gamma } \hat{z}^{o}_{1}$	1	0	0	0	0
${}^{\gamma } \hat{z}^{o}_{2}$	0	1	0	0	0
${}^{\gamma } \hat{z}^{o}_{3}$	0	0	1	0	0
${}^{\gamma } \hat{z}^{o}_{4}$	0	0	0	1	0
${}^{\gamma } \hat{z}^{o}_{5}$	0	0	0	0	1

#### Activation functions for neurons

In the proposed approach, the purelin and softmax activation functions are implemented for each hidden layer and output layer, respectively.

#### Problem formulation for the case study

Once the inputs and outputs of the case study are defined for the training and validation processes, the initial conditions for the optimization problem in the proposal are established.

The upper-level design variable vector limits for the number of MLP hidden layers are defined as $2\le \vec {U}_{\rho _1}\le 5$. The number of nodes in the hidden layer is bounded by $1\le \vec {U}_{\rho _2}\le 5$. For the lower level, the limits associated with the MLP weights and biases are set as $-5\le \vec {L}_{\rho _1}\le 5$ and $-5\le \vec {L}_{\rho _2}\le 5$, respectively.

On the other hand, regarding the weighting values for $F_{up}$, a value of $a_{s_{1}} = 0.1$ is assigned to reflect the importance level attributed to the size of the neural network architecture. In contrast, $a_{s_{2}} = 0.9$ corresponds to the error and validation obtained from $F_{low}$. For $F_{low}$, $a_{low_{1}} = 0.01$ is used to indicate the relevance of the MSE, and $a_{low_{2}} = 0.99$ is assigned to the validation of the proposed approach for each generated architecture.

Then, the optimization problem of the proposed EB-LNAST approach in (13) is solved by DE, as is described in the previous in Algorithm 1. It is important to note that once the model derived from the EB-LNAST approach is obtained, the resulting optimal network is fully specified and does not require the use of differential evolution (DE), as it already includes the trained weights and biases necessary for evaluation on unseen data. However, the bi-level training phase based on DE requires a considerable computational cost. To alleviate this limitation, the use of graphics processing units (GPUs), together with parallel and distributed computing strategies, can substantially reduce execution time while preserving the quality of the obtained solution.

## Experimental results

This section presents the analysis of the results obtained using the EB-LNAST approach in color identification through the ColorPal system. The first section discusses the conditions required for the optimizer to solve the EB-LNAST approach. The second section presents the performance analysis of the EB-LNAST approach, and the final section validates the proposal by comparing it with two fixed neural network architectures: one trained using DE and the other using GD.

### Optimizer conditions

To solve the optimization problem using the EB-LNAST approach, the initial conditions shown in Table 3 are used. The values of the scaling factor *F* and the crossover factor *CR* are proposed based on the suggestion provided in^53^. The population size (*NP*) and the maximum number of generations ($G_{Max}$) are determined through an empirical trial-and-error process, where different values are systematically tested to identify those yielding the best overall performance.

**Table 3:** Initial conditions of the DE algorithm for the EB-LNAST approach.

Parameter	Upper level	Lower Level
*NP*	20	10
$G_{Max}$	10	50
*F*	0.9	[0.3, 0.9]
*CR*	0.9	0.9

To verify and evaluate the behavior of the EB-LNAST approach, this study performs thirty independent executions. The corresponding performance analysis of the proposed approach appears in the following section.

### Performance analysis of the EB-LNAST approach

The complete set of thirty executions of the proposed approach using DE is provided in Table 18 of the appendix. The best executions of such a table are shown in Table 4. The first column indicates the execution number followed by the value of the upper-level objective function $F_{up}$, the first term of the upper level $J_{up_1}$, the lower-level objective function $F_{low}$, the lower level’s first term $J_{{low_1}}$, the lower level’s second term $J_{{low_2}}$, and the $F_{\beta _{test}}$ metric. The $F_{\beta _{test}}$ metric considers one hundred test data points (100 samples), as is described in Section 3.1. It is worth noting that, in all executions of the proposed approach, the generated architecture results in a single hidden layer ($h_{L}=1$) with two nodes ($\eta _{h_1}=2$).

**Table 4:** Results of the fourteen best executions from the EB-LNAST approach.

Executions	$F_{up}$	$J_{up_1}$	$F_{low}$	$J_{{low_1}}$	$J_{{low_2}}$	$F_{\beta \, \text {test}}$
2	−0.591	3	−0.99	0	1	0.91
3	−0.591	3	−0.99	0	1	0.928
6	−0.591	3	−0.99	0	1	0.956
7	−0.591	3	−0.99	0	1	0.93
10	−0.591	3	−0.99	0	1	0.957
11	−0.591	3	−0.99	0	1	0.93
**12**	−0.591	3	−0.99	0	1	**0.972**
14	−0.591	3	−0.99	0	1	0.906
15	−0.591	3	−0.99	0	1	0.954
19	−0.591	3	−0.99	0	1	0.938
22	−0.591	3	−0.99	0	1	0.964
23	−0.591	3	−0.99	0	1	0.946
24	−0.591	3	−0.99	0	1	0.926
29	−0.591	3	−0.99	0	1	0.92

**Fig. 5:**
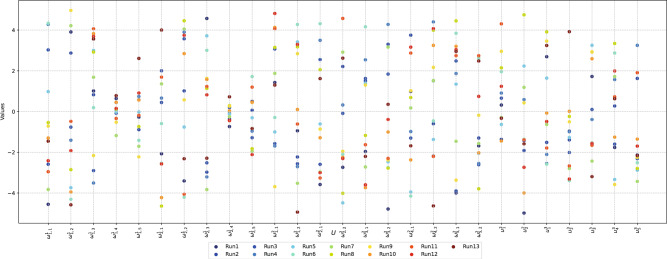
The parallel coordinate plot of the design variables $\vec {U}_\rho$ corresponding to the fourteen best executions with higher-level objective function values $F_{up}=-0.591$ and lower-level objective function value $F_{low}=-0.99$.

The obtained results of the proposed approach yield the same value for the higher-level objective function ($F_{up} = -0.591$) and the lower-level objective function ($F_{low} = -0.99$). The value $F_{low}$ implies that the terms corresponding to the MSE converge to zero ($J_{{low_1}}=0$). The validation metric $F_{\beta _{val}}$ associated with the other term reaches a maximum value ($J_{{low_2}}=1$). This means that no errors are generated during the model’s validation with the proposed approach, implying that the MLP correctly generalizes each pattern from the case study without error, which reflects the outstanding performance of the obtained model. However, despite all executions having the same validation, the difference lies in the trained parameters. The resulting weights and biases show differences, as displayed in Figure 5. So, although the executions converge to the same value for both the higher- and lower-level objective functions, the parameters obtained by the proposed approach exhibit differences. Therefore, the generated architecture has a complex topology with multiple optimal solutions, which confirms the multimodality of the problem.

Since the MLP architecture presents different parameters with the same objective function value, the most appropriate architecture for the case study is selected based on the network’s performance with the test data, with the $F_{\beta \, test}$ metric. In Table 4, the $F_{\beta \, test}$ metric is shown, where a prediction greater than 0.90 is observed in all fourteen executions, confirming that these correctly classify the majority of the solutions found. In particular, the most promising solution (the best for practical purposes) has a value of $F_{\beta \, test}=0.9720$, whereas the worst one has $F_{\beta \, test}=0.9060$. Moreover, all executions present a standard deviation of 0.0195, indicating the variability of the fourteen optimal solutions obtained. In Table 5, the associated variables of the most promising solution with $F_{\beta \, test}=0.9720$ are shown.

**Table 5:** Design variables (DVs) corresponding to the most promising solution with $F_{\beta _{test}}$ = 0.9720.

DVs	Value	DVs	Value	DVs	Value
$\omega _{1,1}^{1}$	4.336559165	$\omega _{1,2}^{1}$	−4.308587149	$u_{1}^{2}$	1.958767935
$\omega _{1,3}^{1}$	0.193691725	$\omega _{1,4}^{1}$	0.122280253	$u_{1}^{3}$	−2.583709719
$\omega _{1,5}^{1}$	−1.415386565	$\omega _{2,1}^{1}$	−0.589810735	$u_{2}^{2}$	−1.360421044
$\omega _{2,2}^{1}$	−4.208863746	$\omega _{2,3}^{1}$	3.005774596	$u_{2}^{3}$	−3.38735932
$\omega _{2,4}^{1}$	0.262737489	$\omega _{2,5}^{1}$	1.718634339	$u_{3}^{3}$	−1.541036076
$\omega _{1,1}^{2}$	−0.292894766	$\omega _{1,2}^{2}$	4.275509663	$u_{4}^{3}$	2.872284614
$\omega _{2,1}^{2}$	4.312974947	$\omega _{2,2}^{2}$	−2.100953911	$u_{5}^{3}$	−2.512897963
$\omega _{3,1}^{3}$	4.162213365	$\omega _{3,2}^{3}$	−2.749309781	$\omega _{5,1}^{2}$	3.842026311
$\omega _{4,1}^{2}$	−4.147006129	$\omega _{4,2}^{2}$	−0.458866391	$\omega _{5,2}^{2}$	2.628829431

**Fig. 6:**
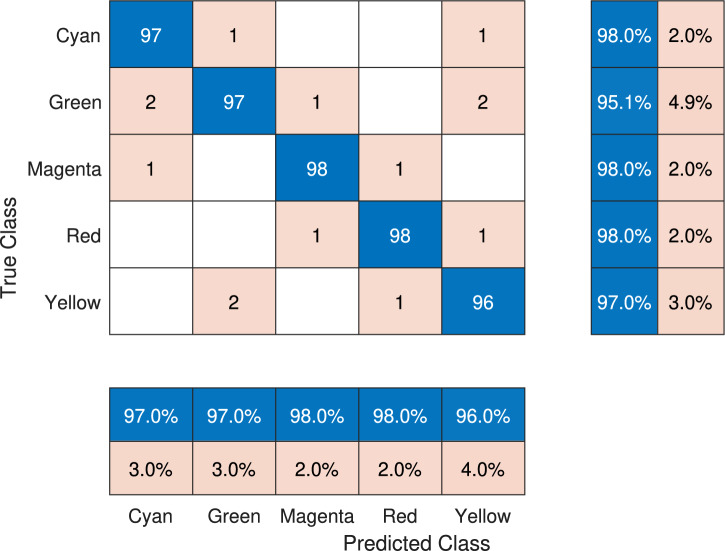
Confusion matrix generated by the EB-LNAST approach corresponding to the best execution with $F_{\beta _{test}}=0.972$.

To analyze the behavior of the most promising solution in its architecture for the case study, a multiclass confusion matrix is shown in Figure 6. The main diagonal of the confusion matrix refers to the total number of correct predictions, i.e., the TPs. The values outside the diagonal represent incorrect predictions, including both FNs and FPs. A summary of such a prediction is presented in the bottom part of the confusion matrix, where the first row of the predicted class box shows the percentage of total TPs for each color, while the second row displays the percentage of errors (sum of FP and FN) obtained for each class. It can be seen that the percentages in the FPs and FNs indices are within the interval $[2, 4]\%$, confirming an acceptable error percentage in color classification within the test set. On the other hand, to the right of the confusion matrix, the prediction index corresponding to the true classes for the proposed approach is shown. It is observed that for each class, the prediction interval is $[95.1, 98.0]\%$, indicating the high precision of the model in correctly classifying each class.

### Comparative analysis with fixed architecture of a DNN

In this section, the proposed EB-LNAST approach is compared with a fixed architecture of a DNN, whose weights and biases are optimized using the DE and GD algorithms. The primary objective is to evaluate the performance of the proposed model against a DNN with multiple hidden layers. Subsequently, the results obtained with DE and GD are analyzed, and a direct comparison is made with the results achieved by the EB-LNAST approach.

Based on the results obtained from the proposed approach in section 4.2, it can be observed that the generated architecture consists of a total of twelve nodes, which are distributed across five nodes in the input and output layers, with one hidden layer ($h_L=1$) containing two nodes in that layer ($\eta _{h_1}=2$). In practice, most neural network architectures are selected based on the designer’s experience and empirical experimentation, testing various configurations to solve the same problem, as in the case of pattern classification, image recognition, regression problems, and/or function approximation^54,55^.

Many of these studies suggest that with a greater number of parameters in the neural network architecture, better performance is likely to be obtained. However, this also comes with an increase in processing time, and many of these studies are exclusively focused on Deep Learning and do not follow an established metric or methodology for architecture selection. This results in the empirical selection of architectures that increase the experimentation time and reduce the repeatability of results due to the trial-and-error process.

Therefore, considering the architecture generated by the EB-LNAST approach, the comparative DNN is formed by a total of 25 nodes grouped among the input layer, output layer, and three hidden layers ($h_L = 3$), with five nodes in each hidden layer ($\eta _{h_1} = 5$, $\eta _{h_2} = 5$, and $\eta _{h_3} = 5$). The comparative DNN exhibits a $108.33\%$ increase in the number of nodes relative to the architecture obtained with the EB-LNAST approach. In other words, the EB-LNAST method achieves a $52\%$ reduction in architectural size compared to the DNN. For this comparison, this new configuration may provide better performance compared to the architecture generated by the proposed approach.

The comparative DNN architecture is optimized using both DE and GD. In the case of the GD algorithm, an increase in the depth of the network leads to problems of vanishing or exploding gradients^56^, especially if the initial conditions are not correctly set. On the other hand, when considering the search for solutions using DE, the increase in the complexity of the architecture implies a greater number of objective function evaluations are required to achieve convergence towards optimal architectures. It is worth mentioning that the same activation functions are employed in the DNN architecture as in the proposed method, namely the purelin function for the hidden layers and the softmax function for the output layer. This design choice ensures a fair comparison by maintaining consistent activation dynamics across models, thereby isolating the effect of other architectural and algorithmic differences. The parameters for GD and DE are shown in Table 6, and those are obtained through experimental observations. In the first and second columns of the table, the parameters used for the GD algorithm are presented, including a maximum number of training epochs $200{,}000$, a Learning Rate $1 \times 10^{-3}$, and weight and bias initialization based on a normal distribution between the interval expressed in Table 6. The third and fourth columns present the parameters used for the DE algorithm. A total of thirty executions are performed for each optimizer (GD and DE).

**Table 6:** Conditions for experiments in the training of the DNN with GD and DE.

GD		DE	
Max epochs	200000	NP	30
Learning Rate	$1 \times 10^{-3}$	$G_{max}$	250
Weight init.	[−0.1, 0.1]	CR	0.6
Bias init.	[−0.1, 0.1]	F	[0.3, 0.9]

Table 19a in the Appendix section presents the total number of executions by using DE and GD in the training of the DNN. Tables 7 and 8 report the best executions in both cases. The test evaluation is also incorporated in the aforementioned tables. A similar presentation of results is provided as in Table 4. It is important to highlight that, during the experimental tests, it is observed that classifications performed using the GD method resulted in output probabilities from the MLP that consistently remained below 0.90. This behavior contrasts with the EB-LNAST and DE approaches applied to DNNs, which produced probabilities approaching 0.99999 and even reaching 1 in some cases. Such a discrepancy indicates a lower level of confidence in the predictions made by the DNN trained with GD. This does not necessarily imply incorrect predictions but rather suggests a reduced likelihood of correct classification according to the model’s internal confidence. To address this issue and accurately reflect the GD-trained model’s behavior on the test dataset, the predicted class is assigned based on the maximum probability output, following the methodology proposed in^57,58^. This allows for a more faithful evaluation of the actual performance of the DNN optimized using GD.

**Table 7:** Performance of the best DE executions in the DNN training, incorporating test evaluation.

Execution	$F_{low}$	$J_{{low_1}}$	$J_{{low_2}}$	$F_{\beta \, test}$
3	−0.99	0	1	0.95
5	−0.99	0	1	0.936
9	−0.99	0	1	0.894
13	−0.99	0	1	0.942
16	−0.99	0	1	0.922
21	−0.99	0	1	0.954
23	−0.99	0	1	0.95
25	−0.99	0	1	0.936
26	−0.99	0	1	0.876
27	−0.99	0	1	0.948
28	−0.99	0	1	0.962
**29**	−0.99	0	1	**0.964**

**Table 8:** Performance of the best execution provided by GD in the DNN training, along with their respective performance on the testing dataset.

Execution	$F_{low}$	$J_{{low_1}}$	$J_{{low_2}}$	$F_{\beta \, test}$
2	−0.99	$3.50\times 10^{-13}$	1	0.946

It is observed that in the case of GD results, only one best solution is found, which presents a $F_{\beta \, test}$ metric of 0.946. Meanwhile, in the case of DE results, twelve solutions with the same training performance are found. Therefore, test performance is used to select the most suitable solution in practice since all executions yield the same or very similar values for the lower-level performance metric $F_{low}$.

The DNN trained with DE achieves the best value of $F_{\beta \, test} = 0.964$, and the rest of the solutions are in the interval $F_{\beta _{test}} \in [0.876, 0.964]$. It is worth noting that higher values closer to one indicate better classification performance. On the other hand, the proposed method yields an $F_{\beta _{test}}$ interval of $[0.9060,\ 0.9720]$. Table 9 presents the 95% confidence intervals for the $F_{\beta \, \text {test}}$ metric achieved by the EB-LNAST and DNN-DE approaches. The obtained confidence intervals suggest that both approaches achieve high performance on the classification task. Nevertheless, the EB-LNAST attains a peak $F_{\beta \, \text {test}}$ value (0.972) larger than DNN-DE (0.964). These outcomes indicate that EB-LNAST provides slightly better peak predictive capability. In addition, the EB-LNAST yields a narrower confidence interval ([0.9267, 0.9501]) than the DNN-DE interval ([0.9191, 0.9532]). This suggests that EB-LNAST not only achieves strong peak performance but also reveals greater consistency and reliability across multiple executions. As a consequence, the EB-LNAST outperforms DNN-DE in terms of both maximum test performance and overall stability, making the solution with the proposed EB-LNAST a more robust one in terms of classification accuracy.

**Table 9:** The 95% confidence interval of the metric $F_{\beta \, test}$.

Confidence interval	
EB-LNAST	[0.9267, 0.9501]
DNN-DE	[0.9191, 0.9532]

**Fig. 7:**
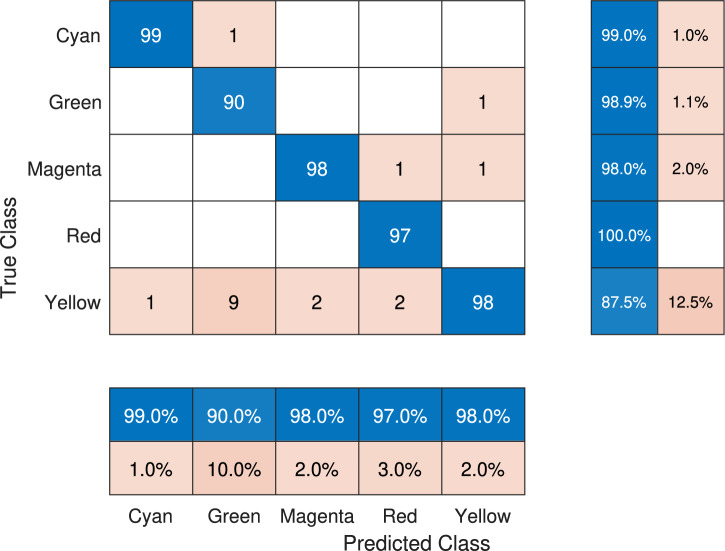
Confusion matrix generated by DE for the DNN architecture corresponding to the best execution with a test $F_{\beta _{test}}$ score of 0.964.

**Fig. 8:**
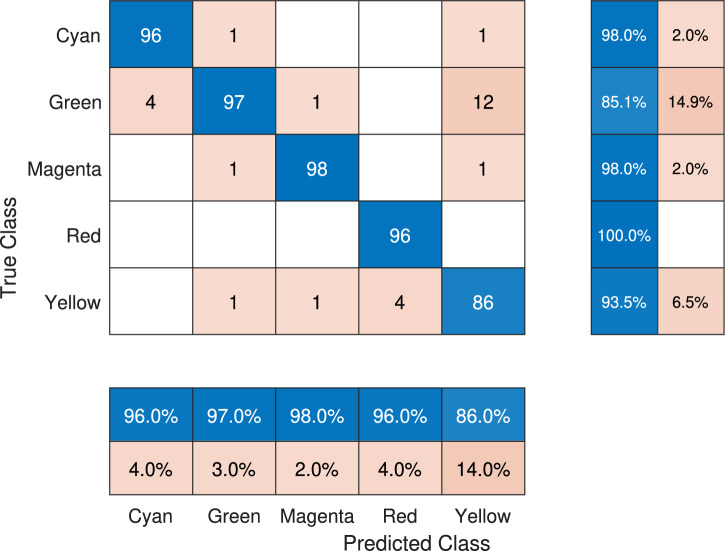
Confusion matrix generated by GD for the DNN architecture corresponding to the best execution with the test $F_{\beta _{test}}$ score of 0.9472.

To observe the distribution of predictions using the DNN trained by GD and DE, the confusion matrices corresponding to those networks are shown in Figure 7 and Figure 8. In the case of DBB-GD, it is observed that there is an error of $14.0\%$ for the yellow class, followed by $4.0\%$ for the red and cyan ones, $3.0\%$ for the green one, and finally $2.0\%$ for the magenta class. This value represents a substantial increase in the error range, which is between $[0.2, 14.0]\%$. Additionally, the prediction index for each class, shown on the right side of the matrix, ranges between $[85.1, 100]\%$. In contrast with DNN-DE, it is observed that the DNN has a $10.0\%$ error when predicting the green class, followed by $3.0\%$ for yellow and red, $2.0\%$ for magenta, and $1.0\%$ for the cyan class. The confusion matrix of DNN-DE shows a prediction error range between $[1,10]\%$. On the other hand, when analyzing the right side of the matrix, it is observed that the correct prediction rate for each class is between $[87.5, 99]\%$.

On the other hand, the inferential statistical analysis is also shown to generalize the obtained conclusion. In particular, the nonparametric statistical analysis provided by the pairwise Wilcoxon signed-rank test is used with the thirty data ($F_{\beta \, test}$) obtained in Tables 18 and 19a, corresponding to the proposed EB-LNAST and the DNN trained by DE, and GD, respectively. Table 10 displays the Wilcoxon test, where two-sided hypothesis and the statistical significance of $\rho = 5\%$ are selected. The columns $R_{+}$ and $R_{-}$ correspond to the sums of ranks computed during the test. Here, $R_{-}$ reflects the instances where samples from the first set outperform those from the second, while $R_{+}$ captures the contrary. The winner is shown in boldface when $p\text {-value} \le \rho$. Based on the Table 10, it is statistically confirmed that EB-LNAST is the best option because it outperforms the results given by both DNN-DE and DNN-GD. The second option is provided by the DNN-DE. The worst result is given by DNN-GD.

**Table 10:** Summary of the inferential statistics for $F_{\beta test}$: EB-LNAST vs. DNNs (DE, GD).

Comparison	${R_{+}}$	${R_{-}}$	p-value
**EB-LNAST** vs DNN-DE	86.5	378.5	0.0019
**EB-LNAST** vs DNN-GD	5.5	459.5	$2\times 10^{-8}$
**DNN-DE** vs DNN-GD	128	337	0.0305

The results obtained indicate that the DNN architectures optimized using DE and GD do not outperform the classification accuracy achieved by the EB-LNAST approach. These findings suggest that arbitrarily increasing the number of hidden layers and nodes in a neural network does not necessarily guarantee improved performance. As mentioned earlier, the designer’s experience can influence the generation of effective classification models. However, the EB-LNAST approach shows that it is possible to identify compact and efficient architectures capable of solving the problem both effectively and reliably. A representative video showing the color classification performed by the model obtained through the EB-LNAST approach is available at https://www.dropbox.com/scl/fo/8b5s6xeyxyd9eexpk761z.

Furthermore, the dataset plays a fundamental role in the design and evaluation of neural architectures. Particularly, due to the multimodal characteristic of the classification problem, it is observed that DE did not encounter difficulties in finding efficient solutions. In contrast, the GD optimizer exhibits lower predictive reliability, as the initial conditions and sensitivity to local minima significantly hinder its performance.

### Comparative analysis with popular machine learning classification algorithms

To enhance the comparative analysis of the EB-LNAST approach in the color classification task, four widely used Machine Learning (ML) classification algorithms implemented using the Scikit-learn library in Python are incorporated: Multi-Layer Perceptron (MLP), K-Nearest Neighbors (KNN), Naive Bayes (NB), and Random Forest (RF). For each algorithm, a hyperparameter optimization process is conducted to assess the effectiveness of the proposed bilevel optimization approach. Then, the comparisons also undergo optimal hyperparameter tuning. For instance, in the case of the MLP, particular attention is given to a comprehensive set of hyperparameters, including not only architectural aspects (number of layers and neurons), weights and bias terms, but also other relevant hyperparameters, such as, activation functions, weight initialization strategies, regularization techniques, gradient-based optimizers, thereby enabling a more comprehensive and fine-grained tuning process.

The hyperparameters of such ML algorithms are tuned by using the hyperparameter optimization from the Scikit-learn tool called “RandomizedSearchCV”, where the general setting of the tool is shown in Table 11 and the hyperparameter search space defined for each model is displayed in Table 12.

**Table 11:** General configuration of the ML algorithms performed in Python using the RandomizedSearchCV tool from the Scikit-learn library.

Configuration	Value
Algorithms	MLP/NB/KNN/RF
Iterations	50
Cross-validation	5
Metric	$F_{\beta }$
Re-training	True
Parallelism	−1
Seed	Variable per run

**Table 12:** Hyperparameter search space used in RandomizedSearchCV tool.

Multi Layer Perceptron (MLP)	
Hidden layers	1 or 2
Hidden layer sizes	Integer values between 2 and 150
Activation function	Relu, tanh, logistic, identity
Solver	Adam, sgd
L2 regularization alpha	Positive continuous values (mean 0.1)
Learning rate init	Values between 0.1 and 0.2
Early stopping	True, False
Epochs (max_iter)	Integer values between 100 and 500
Random Forest (RF)	
Number of estimators	Integer values between 100 and 600
Max depth	Integer values between 3 and 50
Min samples split	Integer values between 2 and 20
Min samples leaf	Integer values between 1 and 20
Max features	Proportion between 0.3 and 0.7
Bootstrap	True, False
Class weight	None, balanced, balanced subsample
Criterion	Gini, cross entropy
K-Nearest Neighbors (KNN)	
Number of neighbors	Integer values between 1 and 60
Weights	Uniform, distance
Metric	Minkowski, euclidean, manhattan, chebyshev
p (for minkowski)	Integer values between 1 and 3
Algorithm	Auto, ball_tree, kd_tree, brute
Leaf size	Integer values between 15 and 60
Naive Bayes (NB)	
Algorithm	Gaussian NB
Var smoothing	Values between $10^{-12}$ and $10^{-6}$

The results of thirty independent executions of the comparative ML algorithms, optimized via hyperparameter tuning, are reported in Table 20 and Table 21 in the appendix. The former table provides the information on the MLP comparison, the latter one for the rest of the ML algorithms (KNN, RF and NB). In addition, to support a general conclusion, Table 14 presents the pairwise Wilcoxon signed-rank test results for the $F_{\beta \text { test}}$ metric, using the same configuration and following the same table description as in previous sections. This table statistically confirms that the proposed EB-LNAST achieves an outstanding performance with respect to KNN, RF and NB, while in the case of MLP, the proposal exhibits a performance drop. In particular, the best $F_{\beta \text { test}}$ scores obtained by KNN, RF, and NB are 0.9272, 0.9265, and 0.8792, respectively. These represent performance reductions of approximately $4.62\%$, $4.68\%$, and $9.55\%$ compared to the proposed method. In the comparative analysis with MLP, the statistical results indicate that the proposed EB-LNAST does not outperform the model based on MLP. Nevertheless, the best $F_{\beta \text { test}}$ score achieved by EB-LNAST (0.972) is only $0.80\%$ lower than the best result obtained by MLP (0.9799). On the other hand, the MLP exhibits architectures with up to two hidden layers (see $h_L$ in Table 20) and variable layer sizes reaching up to 149 neurons per layer (see $\eta _{h_1}$ and $\eta _{h_2}$ in Table 20), which increases the network’s effective complexity as measured by $J_{up_1}$ (4). In contrast, EB-LNAST maintains a fixed and compact structure with a single hidden layer of only two neurons (see $h_L$ and $\eta _{h_1}$ for EB-LNAST), significantly reducing architectural complexity. Then, considering that the proposed method achieves a substantial architectural reduction of $98.51\%$, as indicated by the MLP architecture metric $J_{up_1}$ in Table 20, and that network performance is only marginally affected, the EB-LNAST approach offers a competitive model that significantly enhances the trade-off between architectural complexity and training efficiency by integrating a bi-level architecture search with training parameter optimization.

**Table 13:** Summary of the inferential statistics for $F_{up_1}$: EB-LNAST vs MLP.

**Comparison**	$\mathbf {R_{+}}$	$\mathbf {R_{-}}$	**p-value**
**EB-LNAST** vs MLP	435.0	0.0	$3.7 \times 10^{-9}$

**Table 14:** Summary of the inferential statistics for $F_{\beta test}$: EB-LNAST vs ML algorithms (MLP, KNN, RF and NB).

Comparison	${R_{+}}$	${R_{-}}$	p-value
EB-LNAST vs **MLP**	79.0	386.0	$1.59 \times 10^{-3}$
**EB-LNAST** vs KNN	420.0	45.0	$3.05 \times 10^{-5}$
**EB-LNAST** vs RF	440.0	25.0	$1.68 \times 10^{-6}$
**EB-LNAST** vs NB	450.0	15.0	$7.62 \times 10^{-6}$

On the other hand, to provide a clearer perspective on the computational demands of the proposed approach, the execution times of EB-LNAST and the baseline algorithms are reported. This analysis allows us to contextualize the trade-off between model accuracy and runtime. Table 15 summarizes the average execution time of the evaluated algorithms. It is evident that EB-LNAST requires a substantially higher runtime (approximately 120.7 seconds) compared to conventional algorithms. Among the baselines, Random Forest shows an intermediate runtime (7.58 seconds), whereas MLP, KNN, and NB achieve very low execution times, all below one second. These findings reflect the additional computational effort of EB-LNAST, which results from its bi-level evolutionary optimization strategy. Nevertheless, this effort is strategically invested in obtaining architectures that are not only compact and high-performing but also stable and reproducible properties. Moreover, once trained, these minimal architectures drastically reduce inference time and resource consumption in practical applications with limited computational resources. In this way, the higher computational demand during optimization is balanced by the long-term benefits of efficiency, reproducibility, and practical applicability.

The bi-level optimization stage can be significantly accelerated through parallel and distributed computing strategies, such as GPU-based implementations, which would effectively mitigate the runtime overhead. However, this remains an open direction for future work.

**Table 15:** Average execution time (in seconds) of the compared algorithms.

**Algorithm**	**Average Time (s)**
EB-LNAST	120.7074
MLP	0.3376
RF	7.5807
KNN	0.3644
NB	0.0964

### Comparative analysis with a large dataset

To evaluate the performance of EB-LNAST on a larger and more complex dataset, the Breast Cancer Wisconsin (Diagnostic) Dataset (WDBC) is employed^59^. This dataset consists of 569 samples of digitized images of fine needle aspiration of breast masses classified as either benign (with 357 cases) or malignant (with 212 cases) in an imbalanced class distribution. In the WBDC dataset, ten morphological features of the cell nuclei were extracted: radius, texture, perimeter, area, smoothness, compactness, concavity, concave points, symmetry, and fractal dimension. For each of these base features, three descriptive statistics were computed: the mean, the standard error, and the worst value (defined as the mean of the three largest values). This results in thirty real-valued predictive attributes per sample, in addition to a unique identifier and the diagnostic class label.

Table 22 in appendix presents the results from thirty independent runs of the EB-LNAST approach in the WBDC dataset, using the same DE configuration for color identification, and the dataset is split into 70% for training and 30% for validation and testing to make a fair comparison with the reported results in the literature. Table 16 presents the report summary described by descriptive statistics in the metrics $F_{\beta test}$ and accuracy. It is observed that the proposal achieves the best $F_{\beta test}$ value of 0.9879, with a mean value of 0.9830 and a reduced standard deviation of 0.0031. In addition, the accuracy metric reaches values of 0.9883, 0.9709, and 0.0235, following the same order of comparison. Although of the EB-LNAST approach is formulated to improve the $F_{\beta test}$, the accuracy metric presents a similar value.

**Table 16:** Descriptive statistics of EB-LNAST for the WDBC dataset.

Statistic (Accuracy)	Value	Statistic ($F_{\beta test}$)	Value
Best	**0.9883**	Best	**0.9879**
Worst	0.9006	Worst	0.9758
Mean	0.9709	Mean	0.9830
Std. Dev.	0.0235	Std. Dev.	0.0031

With the purpose of evaluating the effectiveness of the proposed approach on a larger dataset, the best results reported in^60^ with different Machine Learning (ML) algorithms on the WBDC dataset are provided for comparison. The Gated Recurrent Unit-Support Vector Machine (GRU-SVM), Linear Regression (LR), MLP, L1 Nearest Neighbor regularization (L1-NN), L2 Nearest Neighbor regularization (L2-NN), Softmax Regression (SR), and Support Vector Machine (SVM) were the reported ML algorithms in^60^. Table 17 reports the accuracy obtained in the work. It is observed that the proposed EB-LNAST with an accuracy metric of 0.9883 outperforms six of the seven ML algorithms compared with^60^. The best reported results are given by MLP, which obtained an accuracy of $0.9903\%$, slightly superior to the EB-LNAST, with a difference of 0.0021. Nevertheless, this small difference is offset by the remarkably compact architecture obtained with the proposal (a single hidden layer with only five neurons). In particular, the MLP used in^60^ relies on an architecture that is approximately 30000 times larger than the EB-LNAST approach (the obtained model with the proposal approach achieves a $99.66\%$ reduction w.r.t. the MLP in^60^), which requires three hidden layers with five hundred neurons each and uses the ReLU activation function. The results of the proposed approach reflect the particular trade-off defined by the weights assigned to the terms of the upper-level objective function. Alternative weight configurations could emphasize different performance aspects, highlighting the flexibility and competitiveness of the method in adapting to diverse optimization goals, particularly reinforcing its significance for medical data classification tasks.

**Table 17:** Reported results from^60^ on WDBC dataset.

Metric	GRU-SVM	LR	MLP	L1-NN	L2-NN	SR	SVM
Accuracy	93.75%	96.0937%	**99.0384**%	93.5672%	94.7368%	97.6562%	96.0937%

## Conclusions

This work presents an Evolutionary Bi-Level Neural Architecture Search with Training (EB-LNAST) approach for simultaneously optimizing the architecture, weights, and biases of a Multi-Layer Perceptron (MLP) through a bi-level optimization strategy. At the upper level, EB-LNAST generates candidate MLP architectures, while at the lower level, it tunes their weights and biases based on the dataset. The proposed approach is evaluated on a color classification task using a custom experimental setup, as well as on the Wisconsin Diagnostic Breast Cancer (WDBC) dataset.

The results show that the EB-LNAST approach statistically outperforms the evaluation of classification performance in the traditional machine learning algorithms KNN, NB, and RF in the color identification task, as well as advanced models such as GRU-SVM, LR, L1-NN, L2-NN, and SR-SVM on the WDBC dataset.

In the comparison with MLP-based approaches, the proposal achieves a superior predictive performance when the network architecture is excluded in the optimization process, and remains competitive (with a maximum performance decrement of up to $0.99\%$) when hyperparameter optimization extends beyond weights, and bias terms, to include additional factors such as architectural aspects (number of layers and neurons), activation functions, weight initialization strategies, regularization techniques, and gradient-based optimizers. In addition, the model obtained with the proposed approach achieves up to a $99.66\%$ reduction compared to MLP-based methods.

The results show that the EB-LNAST approach consistently identifies compact architectures while achieving competitive or superior predictive performance compared with machine learning classification algorithms for the color classification task, and the results obtained in the reported literature for the WDBC dataset.

By effectively exploring the architecture space and the training parameters in a bi-level framework, EB-LNAST identifies minimal yet high-performing networks, offering a robust alternative to traditional empirical design strategies. It also eliminates the need for exhaustive experimentation, resulting in significant time savings and more consistent outcomes.

Future work involves the application extension of EB-LNAST to other neural network architectures (e.g., convolutional neural networks or recurrent neural networks) and application domains with experimental evidence beyond color classification, for instance, optimal neural architectures for interpreting movement intention in a robotic platform, where the goal is to characterize and classify surface electromyographic (sEMG) signals from human upper limbs, enabling accurate translation of intended movements into robotic actions.

## Data Availability

Data will be made available upon request from M.A.G.-O. or M.G.V.-C.

## Appendix

This section presents the complete results for the EB-LNAST approach and the DNN training with DE and GD, as well as models built by other machine learning algorithms. This also presents the results of the proposal with a large dataset.

**Table 18:** Performance evaluation of the EB-LNAST results in thirty executions, with the corresponding testing phase included.

Execution	$F_{up}$	$J_{up_1}$	$F_{low}$	$J_{1_{low}}$	$J_{2_{low}}$	$F_{\beta \, test}$
1	-0.578	3	-0.976	0	0.986	0.930
2	-0.591	3	-0.990	0	1.000	0.910
3	-0.591	3	-0.990	0	1.000	0.928
4	-0.578	3	-0.976	0	0.986	0.930
5	-0.566	3	-0.962	0	0.971	0.920
6	-0.591	3	-0.990	0	1.000	0.956
7	-0.591	3	-0.990	0	1.000	0.930
8	-0.565	3	-0.962	0	0.971	0.908
9	-0.578	3	-0.976	0	0.986	0.942
10	-0.591	3	-0.990	0	1.000	0.958
11	-0.591	3	-0.990	0	1.000	0.930
**12**	-0.591	3	-0.990	0	1.000	**0.972**
13	-0.578	3	-0.976	0	0.986	0.900
14	-0.591	3	-0.990	0	1.000	0.906
15	-0.591	3	-0.990	0	1.000	0.954
16	-0.553	3	-0.948	0	0.957	0.886
17	-0.566	3	-0.962	0	0.971	0.964
18	-0.578	3	-0.976	0	0.986	0.900
19	-0.591	3	-0.990	0	1.000	0.938
20	-0.578	3	-0.976	0	0.986	0.936
21	-0.541	3	-0.935	0	0.944	0.830
22	-0.591	3	-0.990	0	1.000	0.964
23	-0.591	3	-0.990	0	1.000	0.946
24	-0.591	3	-0.990	0	1.000	0.926
25	-0.566	3	-0.962	0	0.971	0.914
26	-0.578	3	-0.976	0	0.986	0.920
27	-0.578	3	-0.976	0	0.986	0.934
28	-0.565	3	-0.962	0	0.971	0.880
29	-0.591	3	-0.990	0	1.000	0.920
30	-0.565	3	-0.961	0	0.971	0.910

**Table 19:** Obtained results of the DNN’s training using DE and GD including testing performance.

DE					GD.				
Execution	$F_{low}$	$J_{1_{low}}$	$J_{2_{low}}$	$F_{\beta \, test}$	Execution	$F_{low}$	$J_{1_{low}}$	$J_{2_{low}}$	$F_{\beta \, test}$
1	-0.701	0.400	0.712	0.742	1	-0.948	$4.20\times 10^{-12}$	0.958	0.844
2	-0.715	0.400	0.726	0.762	**2**	-0.990	$3.50\times 10^{-12}$	1.000	**0.946**
3	-0.990	0.000	1.000	0.950	3	-0.905	$3.84\times 10^{-12}$	0.914	0.834
4	-0.935	0.000	0.944	0.810	4	-0.919	$4.12\times 10^{-12}$	0.928	0.870
5	-0.990	0.000	1.000	0.936	5	-0.947	$5.07\times 10^{-12}$	0.957	0.890
6	-0.921	0.000	0.930	0.816	6	-0.756	$9.14\times 10^{-12}$	0.763	0.648
7	-0.976	0.000	0.986	0.906	7	-0.921	$2.73\times 10^{-12}$	0.930	0.808
8	-0.976	0.000	0.986	0.930	8	-0.893	$3.32\times 10^{-12}$	0.902	0.832
9	-0.990	0.000	1.000	0.894	9	-0.893	$4.30\times 10^{-12}$	0.902	0.828
10	-0.686	0.400	0.697	0.628	10	-0.920	$3.72\times 10^{-12}$	0.929	0.838
11	-0.682	0.400	0.693	0.724	11	-0.920	$3.02\times 10^{-12}$	0.929	0.868
12	-0.948	0.000	0.958	0.856	12	-0.962	$3.05\times 10^{-12}$	0.971	0.928
13	-0.990	0.000	1.000	0.942	13	-0.934	$3.01\times 10^{-12}$	0.944	0.864
14	-0.722	0.400	0.733	0.776	14	-0.948	$2.70\times 10^{-12}$	0.958	0.918
15	-0.932	0.000	0.942	0.828	15	-0.920	$3.05\times 10^{-12}$	0.929	0.840
16	-0.990	0.000	1.000	0.922	16	-0.822	$1.45\times 10^{-11}$	0.830	0.686
17	-0.962	0.000	0.971	0.896	17	-0.693	$5.52\times 10^{-12}$	0.700	0.640
18	-0.722	0.400	0.733	0.772	18	-0.687	$5.53\times 10^{-12}$	0.694	0.622
19	-0.947	0.000	0.957	0.884	19	-0.749	$2.94\times 10^{-12}$	0.757	0.744
20	-0.722	0.400	0.733	0.748	20	-0.921	$4.14\times 10^{-12}$	0.930	0.880
21	-0.990	0.000	1.000	0.954	21	-0.714	$9.05\times 10^{-12}$	0.721	0.628
22	-0.947	0.000	0.957	0.826	22	-0.759	$2.07\times 10^{-12}$	0.766	0.764
23	-0.990	0.000	1.000	0.950	23	-0.699	$1.12\times 10^{-11}$	0.707	0.654
24	-0.712	0.400	0.723	0.778	24	-0.948	$4.20\times 10^{-12}$	0.958	0.844
25	-0.990	0.000	1.000	0.936	25	-0.645	$8.64\times 10^{-12}$	0.652	0.590
26	-0.990	0.000	1.000	0.876	26	-0.948	$3.26\times 10^{-12}$	0.958	0.888
27	-0.990	0.000	1.000	0.948	27	-0.692	$9.14\times 10^{-12}$	0.699	0.584
28	-0.990	0.000	1.000	0.962	28	-0.847	$4.12\times 10^{-12}$	0.856	0.786
**29**	-0.990	0.000	1.000	**0.964**	29	-0.922	$3.64\times 10^{-12}$	0.931	0.822
30	-0.976	0.000	0.986	0.878	30	-0.768	$6.93\times 10^{-12}$	0.775	0.692

**Table 20:** Obtained results of the models with MLP and the EB-LNAST approach.

MLP.						EB-LNAST.				
Run	$F_{\beta \,\text {test}}$	$h_L$	$\eta _{h_1}$	$\eta _{h_2}$	$J_{{up}_{1}}$	Run	$F_{\beta \,\text {test}}$	$h_L$	$\eta _{h_1}$	$J_{{up}_{1}}$
1	0.8590	1	149	0	150	1	0.930	1	2	3
2	0.9181	1	112	0	113	2	0.910	1	2	3
3	0.9720	2	141	22	165	3	0.928	1	2	3
4	0.9184	2	38	104	144	4	0.930	1	2	3
**5**	**0.9799**	**2**	**98**	**101**	**201**	5	0.920	1	2	3
6	0.9480	2	9	144	155	6	0.956	1	2	3
7	0.8989	1	108	0	109	7	0.930	1	2	3
8	0.9461	2	123	46	171	8	0.908	1	2	3
9	0.9740	2	108	68	178	9	0.942	1	2	3
10	0.9581	2	127	145	274	10	0.958	1	2	3
11	0.9438	2	17	110	129	11	0.930	1	2	3
12	0.9401	2	52	100	154	**12**	**0.972**	**1**	**2**	**3**
13	0.9496	2	72	82	156	13	0.900	1	2	3
14	0.9760	2	76	147	225	14	0.906	1	2	3
15	0.9779	1	79	0	80	15	0.954	1	2	3
16	0.9759	1	24	0	25	16	0.886	1	2	3
17	0.9800	2	110	149	261	17	0.964	1	2	3
18	0.9740	1	70	0	71	18	0.900	1	2	3
19	0.9520	1	44	0	45	19	0.938	1	2	3
20	0.9739	1	102	0	103	20	0.936	1	2	3
21	0.9238	1	48	0	49	21	0.830	1	2	3
22	0.9582	2	130	75	207	22	0.964	1	2	3
23	0.9780	2	44	82	128	23	0.946	1	2	3
24	0.9761	1	82	0	83	24	0.926	1	2	3
25	0.9639	1	73	0	74	25	0.914	1	2	3
26	0.9679	1	132	0	133	26	0.920	1	2	3
27	0.9367	1	134	0	135	27	0.934	1	2	3
28	0.8485	1	121	0	122	28	0.880	1	2	3
29	0.9424	2	91	44	137	29	0.920	1	2	3
30	0.9661	1	72	0	73	30	0.910	1	2	3

**Table 21:** Obtained results of the models with KNN, RF, and NB across thirty runs.

Run	$F_{\beta \,\text {test}}$		
	KNN	RF	NB
1	0.8842	0.8808	**0.8792**
2	0.9187	0.8915	**0.8792**
3	**0.9272**	0.8974	**0.8792**
4	0.9187	0.8874	**0.8792**
5	0.8824	0.8891	**0.8792**
6	0.9187	0.9128	**0.8792**
7	0.9207	0.8918	**0.8792**
8	0.9091	0.8579	**0.8792**
9	0.8812	0.8792	**0.8792**
10	0.8735	0.8942	**0.8792**
11	0.8803	0.8873	**0.8792**
12	0.8937	0.8990	**0.8792**
13	0.9187	0.8816	**0.8792**
14	0.8949	0.8943	**0.8792**
15	0.8692	0.9073	**0.8792**
16	0.8745	0.8796	**0.8792**
17	0.9187	0.8682	**0.8792**
18	0.8862	0.8669	**0.8792**
19	0.8729	0.9150	**0.8792**
20	0.9030	0.8739	**0.8792**
21	0.8729	0.8816	**0.8792**
22	0.8729	0.8629	**0.8792**
23	0.9030	0.8727	**0.8792**
24	0.8977	**0.9265**	**0.8792**
25	0.9013	0.8727	**0.8792**
26	0.8750	0.8832	**0.8792**
27	0.8637	0.8796	**0.8792**
28	0.8677	0.8816	**0.8792**
29	0.8724	0.8816	**0.8792**
30	**0.9272**	0.8646	**0.8792**

**Table 22:** Obtained results with the EB-LNAST for the WBDC dataset.

$F_{up}$	$J_{up_1}$	$F_{low}$	$J_{low_1}$	$J_{low_2}/F_{\beta test}$	Accuracy
-0.8180	6	-0.9695	0.25	0.9818	0.9006
-0.7837	10	-0.9718	1.11e-14	0.9816	0.9415
-0.7919	9	-0.9708	0.125	0.9818	0.9181
-0.8191	6	-0.9707	0.125	0.9817	0.9649
-0.7747	11	-0.9719	0	0.9817	0.9591
-0.8191	6	-0.9708	0.125	0.9818	0.9357
-0.8111	7	-0.9719	0	0.9817	0.9649
-0.8256	**6**	-0.9780	0	**0.9879**	0.9766
-0.8331	4	-0.9661	0	0.9758	0.9298
-0.7918	9	-0.9707	0.125	0.9817	0.9415
-0.8094	7	-0.9700	0.125	0.9811	0.9825
-0.8060	**8**	-0.9763	0.125	0.9874	**0.9883**
-0.8196	6	-0.9713	0	0.9811	0.9825
-0.7980	**9**	-0.9776	0	0.9874	**0.9883**
-0.8434	**4**	-0.9776	0	0.9874	**0.9883**
-0.7993	8	-0.9689	0.25	0.9812	0.9825
-0.8343	**5**	-0.9776	0	0.9874	**0.9883**
-0.7718	11	-0.9686	0.25	0.9810	0.9825
-0.8434	**4**	-0.9775	1.11e-14	0.9873	**0.9883**
-0.7991	8	-0.9686	0.25	0.9810	0.9825
-0.8253	**6**	-0.9776	0	0.9874	**0.9883**
-0.8196	6	-0.9713	0	0.9811	0.9825
-0.8288	5	-0.9714	0	0.9812	0.9825
-0.8196	6	-0.9713	0	0.9811	0.9825
-0.8106	7	-0.9714	0	0.9812	0.9825
-0.8162	**7**	-0.9776	0	0.9874	**0.9883**
-0.8288	5	-0.9714	0	0.9812	0.9825
-0.7913	9	-0.9702	0.125	0.9812	0.9825
-0.8230	**6**	-0.9751	0.25	0.9874	**0.9883**
-0.8197	6	-0.9714	0	0.9812	0.9825
